# Fluocinolone Acetonide Enhances Anterograde Mitochondria Trafficking and Promotes Neuroprotection against Paclitaxel-Induced Peripheral Neuropathy

**DOI:** 10.1021/acschemneuro.3c00218

**Published:** 2023-05-11

**Authors:** Arjun Prasad Tiwari, Lee Ji Chao Tristan, Bayne Albin, In Hong Yang

**Affiliations:** † Center for Biomedical Engineering and Science, Department of Mechanical Engineering and Engineering Science, University of North Carolina at Charlotte, Charlotte, North Carolina 28223, United States; ‡ Department of Biomedical Engineering, National University of Singapore, Singapore 119077, Singapore; § School of Medicine, University of Western Australia, Perth, Western Australia 6009, Australia

**Keywords:** fluocinolone acetonide, mitochondrial trafficking, two-color staining, paclitaxel-induced peripheral neuropathy, neuroprotection

## Abstract

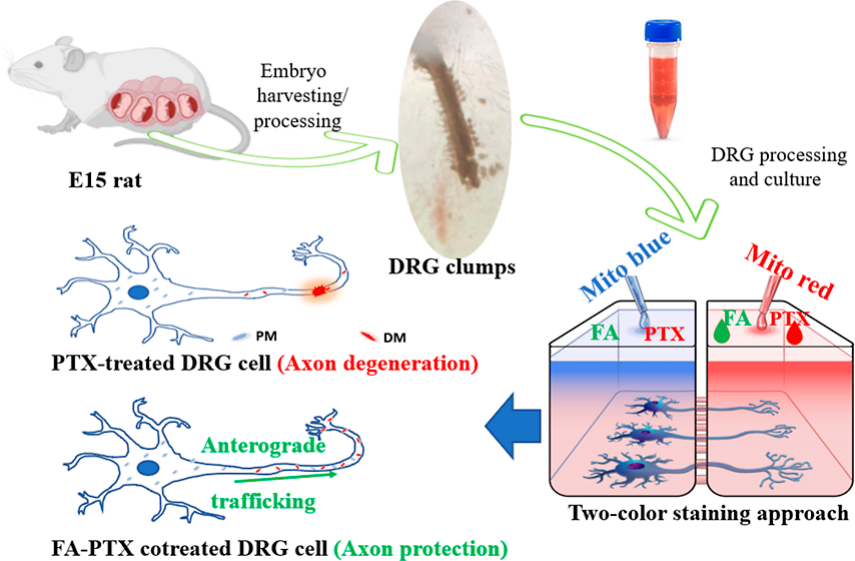

Paclitaxel (PTX)-induced peripheral neuropathy (PIPN) is a debilitating health condition which is a result of degeneration of peripheral nerves found in extremities. Currently, there are no established treatment methods that can prevent or protect from PIPN. Fluocinolone acetonide (FA) has been recently identified as a potential candidate for protection from PIPN. However, the fundamental mechanism of action is still unknown. In this study, we showed that enhanced anterograde mitochondrial movement in dorsal root ganglion (DRG) cells has a major role in FA-mediated neuroprotection in PIPN. In this study, cells were treated with PTX or FA along with their combination followed by mitochondrial fluorescence staining. Somal (proximal) and axonal (distal) mitochondria were selectively stained using a microfluidic compartmentalized chamber with different MitoTrackers blue and red, respectively, which we termed, the two-color staining approach. Results revealed that axons were protected from degeneration by the PTX effect when treated along with FA. PTX exposure alone resulted in low mitochondrial mobility in DRG cells. However, cotreatment with PTX and FA showed significant enhancement of anterograde trafficking of somal (proximal) mitochondria to distal axons. Similarly, cotreatment with FA restored mitochondrial mobility significantly. Overall, this study affirms that increasing mitochondrial recruitment into the axon by cotreatment with FA can be a worthwhile strategy to protect or prevent PIPN. The proposed two-color staining approach can be extended to study trafficking for other neuron-specific subcellular organelles.

## 1 Introduction

Cancer is the second leading cause of death after cardiovascular diseases and is now a global health problem. In 2019, around 2 million people have been diagnosed with cancer in the United States alone.^1^ Chemotherapy has been considered to be an effective tool in hindering cancer progression and increasing the patient survivability rate.^2^ Chemotherapy treatment is effective in eliminating the division of cancer cells while being able to target numerous locations. However, many adverse health effects come along with introducing chemotherapy drugs in patients.^3^ Peripheral neuropathy is one of the many side effects caused by a substantial number of chemotherapy treatments including taxanes, vinca alkaloids, platinum-based antineoplastic agents, proteasome inhibitors (bortezomib), and so forth.^4^ The clinical manifestation of peripheral neuropathy is pain sensation, numbness, and tingling and unusual sensations such as mechanical and thermal allodynia and hyperalgesia.^5^ The progression of peripheral neuropathy results in dosage delay, dose reduction, substitutions, and cessation of chemotherapy in patients who develop intolerable neuropathy or functional impairment.^6,7^ Paclitaxel (PTX) is a widely used anticancer drug for various solid tumors, where microtubules are immobilized by PTX action and cause cell death.^8,9^ The prevalence of PTX-induced peripheral neuropathy (PIPN) is estimated to range from 59 to 87% in cancer patients and survivors.^10^ However, there are not any established drug candidates that can either prevent or protect those suffering from PIPN.

PIPN is predominately a sensory neuropathy in which symptoms can be acute or emerge after chemotherapy treatment following weeks or months of drug courses.^11^ Symptoms develop usually in the extremities such as the feet and hands and are commonly called gloves and stocking patterns.^12,13^ PIPN is characterized as axonal neuropathy where small, nonmyelinated, and longer axons degenerate first.^14^ Nonetheless, the underlying mechanisms of the pathobiology of PIPN have not been fully understood. PIPN-associated pain management is often done with common analgesic drugs, but outcomes are far from satisfactory.^15^

Fluocinolone acetonide (FA), an FDA-approved synthetic hydrocortisone derivative, has been recently identified as a neuroprotective agent and has been found to cause a decrease in axonal degeneration from CIPN/PIPN.^16,17^ Our past works in animal studies showed that FA cotreatment protected the sensory intraepidermal fibers which are the most common nerves that experience degeneration in PIPN. Additionally, neuroprotection was confirmed by significantly improving the clinical symptoms of PIPN, i.e., thermal and mechanical withdrawal latency. In a separate report, FA has shown potency for neuroprotection in retinal neuropathy in a rat model as measured by retinogram and histologic analysis.^17,18^ FA-treated retinopathy rats locally demonstrated significantly fewer microglial cells than the nontreated ones. In general, steroids are believed to reduce inflammatory cytokines, which have been used in controlling inflammation and pain in diabetic neuropathy.^19^ Increasing evidence suggests that dysregulation of mitochondria caused by chemotherapies results in loss of mitochondrial potential, reduction in mitochondrial biogenesis, and impairment of mitochondrial transport in axons that developed painful neuropathy mostly in distal body parts.^20^ Other reports have emphasized the increased mobilization of the mitochondria from the cell body to the axon as a neuroprotective strategy for the demyelinated axons or mechanically injured axons.^21^ However, the consequence of neuroprotection by the FA in PIPN is poorly understood. The abundance of adenosine triphosphate (ATP) production levels by sensory neurons under FA-PTX treatment in in vitro conditions and preservation of small sensory axons in rats treated with FA-PTX raises the possibility that increased mitochondrial population and preferential mobility to distal axons may have a role for neuroprotection in PIPN.

**Scheme 1:**
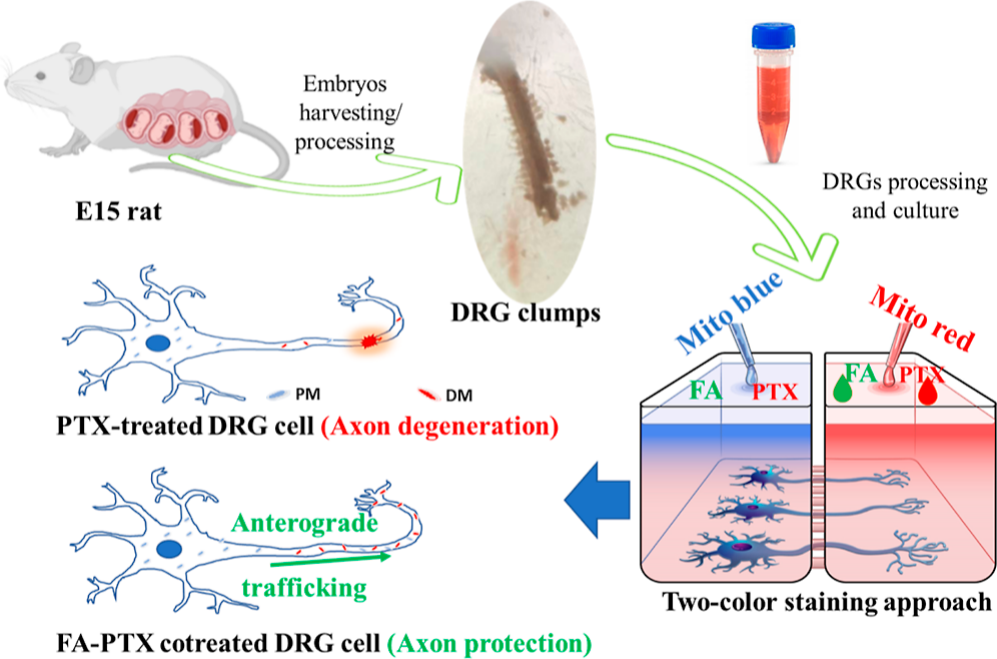
Process Showing Major Experimental Steps in This Study The illustration of the E15 rat was created by Biorender, Scientific Image and Illustration Software.

In this study, the effect of PTX on the mitochondria of dorsal root ganglion (DRG) cells was studied. Furthermore, FA’s role in recruiting mitochondria from the cell body into axons and subsequent neuroprotection in FA-PTX-treated DRG cells was studied as shown in Scheme 1. Integration of a compartmentalized neuronal culture, a two-color mitochondrial staining approach, and time-lapse imaging were used to study the mitochondrial position and trafficking over time. Recently, a compartmentalized neuron culture system has gained immense interest due to its ability to provide spatiotemporal control over the neuronal segments and permit specific compartmentalized testing.^22^ Labeling of subcellular parts with specific markers in the compartmentalized culture system allows for specific localization and manipulation.^23,24^ A two-color mitochondrial staining approach was used to differentiate between somal (proximal) and axonal (distal) mitochondria and trafficking. We used this platform to test a hypothesis that FA engages in transporting mitochondria from the soma to the distal axons which provide neuroprotection against PIPN.

## 2 Results

### 2.1 Optimization Study of PTX and/or FA-PTX Doses for DRG Cell Culture

**Figure 1:**
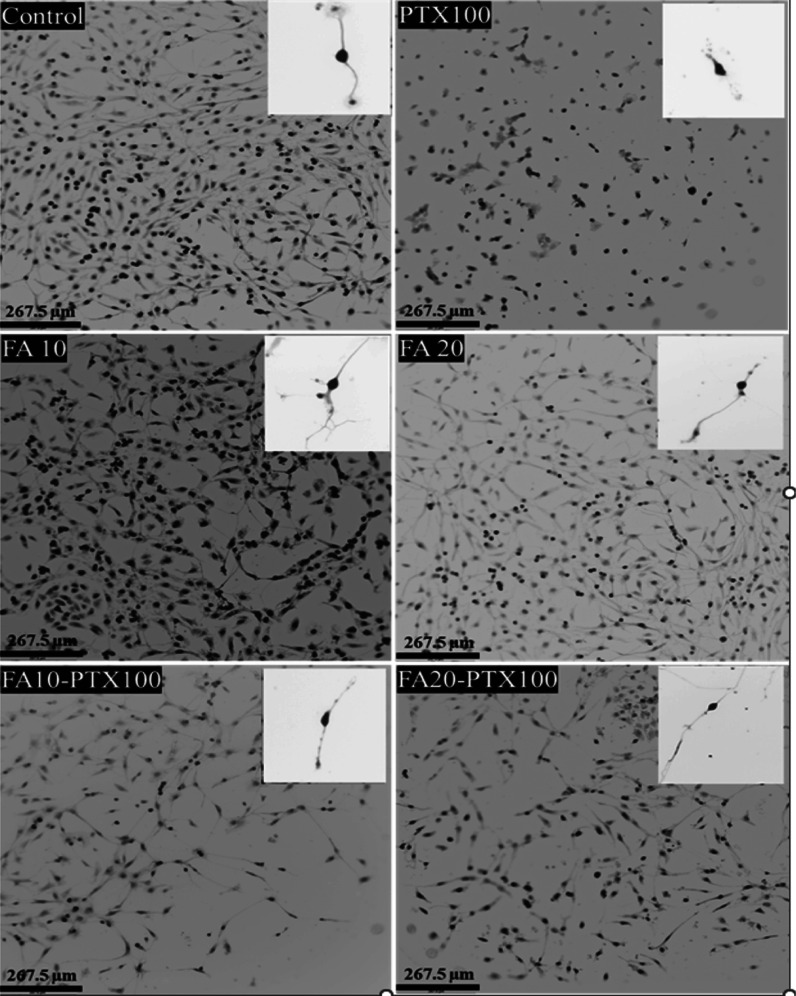
Fluorescence images of calcein-AM-stained DRG cells. DRG cells were exposed to PTX, FA, or FA-PTX of different concentrations for 24 h before staining.

For this study, we cultured DRG cells in a 96-well plate where cells were treated with PTX, FA, and PTX-FA of different concentrations for 24 h as shown in Table 1, and the response of axon length was evaluated by microscopy and further processed by ImageJ software. Results showed that PTX treatment to cells caused the axon length to gradually be reduced with increasing concentrations (Table 1). However, cotreatment with FA resulted in the axon length being increased significantly. For instance, PTX100 had an average axon length of 46.2 ± 21.4 μm which reached 143.6 ± 39.7 and 149.1 ± 30.3 μm when PTX100 was cotreated with FA 10 and FA20, respectively (Table 1). FA10 and FA20 showed enhanced axon growth, which is comparable to that of the control group. It is also critical to note that the axons were found to be shorter at FA100 (140.6 ± 18.7 μm) compared to FA10 and FA20, indicating that axonal growth was affected by the FA concentration. Still, the axon lengths were significantly higher for the cells cotreated with FA100–PTX100 (113.3 ± 34.2 μm) than the PTX100-only-treated (46.2 ± 21.4 μm) cells. The fluorescence images taken of the cells with various drug combinations are shown in Figure 1. PTX100 showed apparent disintegration of the axons; however, axons were still integrated (Figure 1) and found to be protected from degeneration after FA cotreatment regardless of the concentration. This result suggests FA’s ability to retain axonal integrity from the stress induced by PTX. The concentrations of FA and PTX of 20 nM and 100 nM, respectively, were set for the cotreatment in DRG cells for further experiments. The effect of the 5-day exposure to FA-PTX combination on DRG cells was also studied to study the further validity of prior data. The corresponding images of axon length are shown in Figure S1. All drug compositions, except PTX, showed increased axon lengths. For instance, FA-PTX-treated samples had 687.12 ± 120.7 μm long axons on average, while control cells had 733.2 ± 125.9 μm (*P* > 0.05). The effect of the post-FA treatment on PTX-treated cells was also studied at 24 h point. FA was treated for 1 h following PTX subjected to the cells. Results showed that post-FA exposure retained the axonal integrity and protection against the PTX effect (Figure S2C). The post-FA axonal length (147.2 ± 43.1 μm) was comparable to the cotreated condition (149.1 ± 30.3 μm). This preliminary data suggests that post-FA treatment may have the potential to reverse PIPN.

**Table 1:** Axon Length Data in Response to FA, PTX, or Combination^a^

	control	PTX 20	PTX 50	PTX 100	PTX 200	FA 10	FA 20	FA 100	FA10-PTX100	FA20-PTX100	FA100-PTX100
average axon length (μm)	169.5	136.3	121.2	46.2	38.2	170.2	174.0	140.6	143.6	149.1	113.3
min (μm)	126.6	112.4	89.8	35.3	22.54	137.3	119.1	114.6	95.4	107.5	84.1
max (μm)	234.4	160.6	178.9	90.7	64.5	225.8	236.3	164.6	207.2	183.3	171.4
standard deviation (μm)	45.4	17.7	34.9	21.4	19.7	35.3	39.6	18.7	39.7	30.3	34.2

a The axon length of the samples was measured after 24 h of treatment with drugs. 1000 cells were cultured on the PDL/laminin-coated glass-bottom 96-well plates for 24 h followed by designated drug treatment for another 24 h before staining. 50 cells from each well in triplicate samples for each group were used for the axon length measurement. The number that comes along with the drug represents the concentration of the respective drug in nanomoles (nM).

### 2.2 Study of the Effect of FA-PTX Cotreatment on Axonal Mitochondria Using a Two-Color Staining System

**Figure 2:**
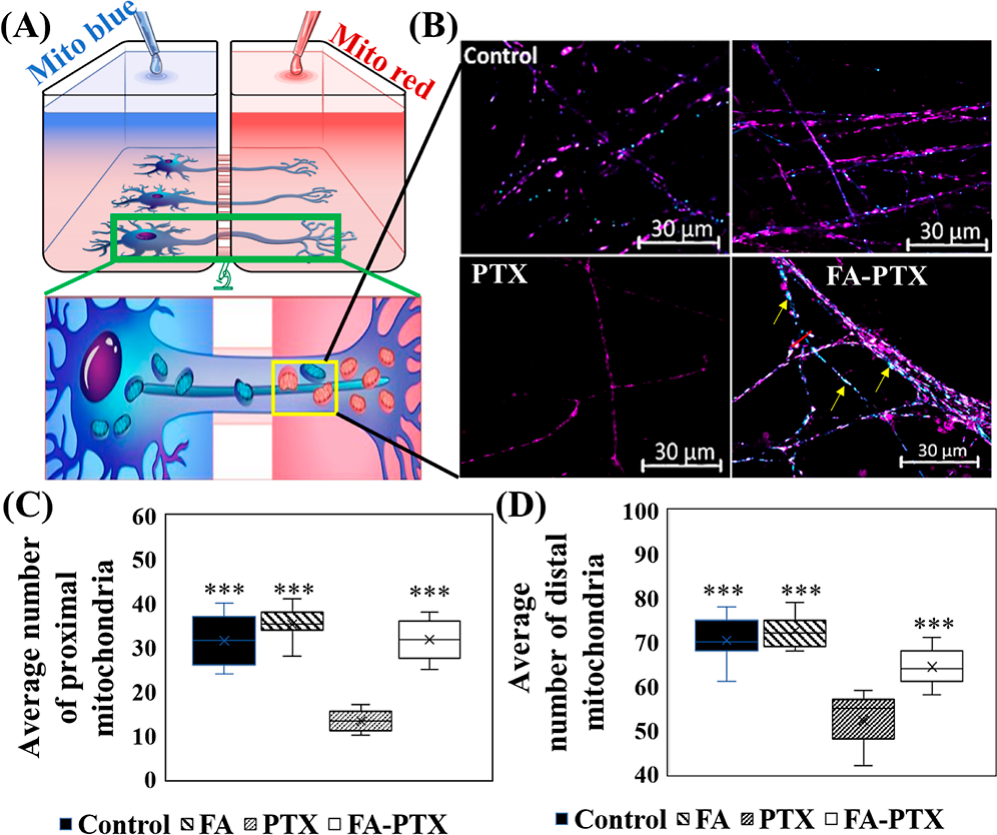
(A) Schematic illustration of the two-color staining approach in a compartmentalized chamber. The inset is the illustration of a high-resolution microscopy image of the neurons in the compartmentalized chamber. The blue and red objects in the neurons are the somal (proximal) and axonal (distal) mitochondria stained specifically by blue and red MitoTracker, respectively. (B) Representative images of the mitochondria distribution in the axonal chamber with each treatment. (C) Average number of proximal mitochondria observed in the axonal chamber and (D) average number of distal mitochondria observed in the axonal chamber. Both mitochondrial populations decreased in PTX-treated axons, while FA-PTX treatment led to a robust recovery. The data represent mitochondria imaged in 10,000 μm^2^ in the distal axon chamber; error bars = S.D. The average number of mitochondria was acquired in triplicate samples for each group. Three frames were used for each sample. Significance was determined for mitochondria between the PTX-treated group and others via a two-tailed *t*-test Excel 2022 (***p < 0.001).

A two-compartment microfluidic device was used as a platform for the study of subcellular mitochondria specific to axons and cell bodies. Soma and axonal mitochondria and their trafficking were evaluated using the two-color staining approach on the fifth day of cell culture treated with PTX, FA, or combination as shown in the schematic (Figure 2A). CIPN was characterized by poor axonal health caused by mitochondrial movement impairment.^25^ Therefore, the recruitment of healthier mitochondria together with rescuing the axonal mitochondria are key criteria to overcome peripheral neuropathy. Figure 2B demonstrates mitochondria in axon chambers in response to various compositions. All samples including the FA-treated ones had dual staining, although they varied by intensity. White-colored objects in the image could be fused mitochondria (Figure 2B, arrows) may be attributed to the fusion of the blue and red-stained mitochondrion during the trafficking process.

As noted in Figure 2C,D, the number of the proximal and distal mitochondria found in the axonal chambers varied upon the treatment with PTX, FA, or FA-PTX. The PTX-only treatment had 13 ± 3 proximal and 53 ± 6 distal mitochondria. With the cotreatment of FA-PTX, the number of proximal and distal mitochondria increased to 32 ± 6 and 65 ± 5, respectively (***P < 0.001). FA induced the highest number of proximal mitochondria trafficking to the axonal chamber compared to other samples (Figure 2C,D). Furthermore, the effect of cotreatment of FA-PTX after 48 h on mitochondria was studied close to the axonal channels. The result showed that there was 30 ± 6 PM (∼75%) in the channels close to the axonal chamber while only 11 ± 5 DM (∼27%) (Figure S3). These results suggest that FA cotreatment with PTX enhanced mitochondrial recruitment from the proximal region toward distal axons.

### 2.3 Effect of FA-PTX on Mitochondrial Transport and Mobility

**Figure 3:**
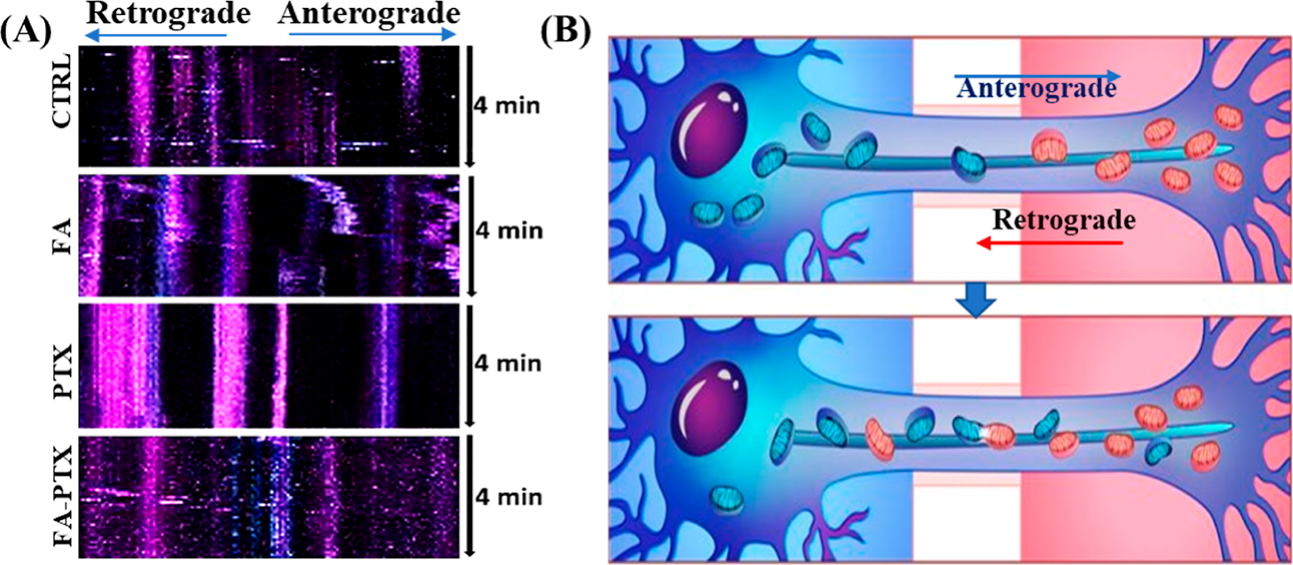
(A) Representative kymographs under different treatment conditions. Anterograde trafficking is represented in the left-to-right direction. The time domain is specified in the top-down *y*-axis; in this work, the mitochondrial movement was graphed for 4 min. Anterograde trafficking is represented in the left-to-right direction, while retrograde trafficking is represented in the opposite direction. Stationary proximal and distal mitochondria are represented in columns in blue and red coloration, respectively, and (B) schematic illustration of the anterograde and retrograde movement of the mitochondria.

**Figure 4:**
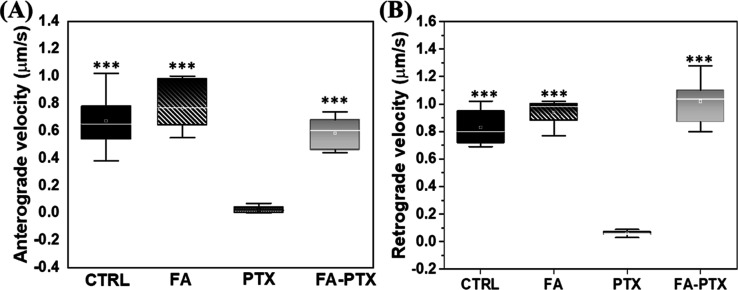
(A) Anterograde mitochondrial velocity and (B) retrograde mitochondria velocity under each treatment in DRG cell culture. The data represent the respective average values from 20–30 mitochondria; error bars = S.D.; significance was determined between the PTX-treated group and others via two-tailed *t*-test Excel 2022 (***p < 0.001).

As shown in Figure 3A, we have used a kymograph to plot the space-time effect of FA and PTX on mitochondrial mobility over a 4-min time period. Results indicate that FA facilitated the mitochondrial anterograde movement at a superior rate (Figure 3A). Cells subjected to PTX showed mitochondria with neither anterograde nor retrograde mobility as indicated by the columns present in kymographs. Figure 3B illustrates mitochondrial anterograde and retrograde movement. Interestingly, the FA-PTX-treated sample restored the anterograde and retrograde mitochondrial mobility to the control level (Figure 4A,B). The representative video showing the movement of mitochondria on the FA-PTX sample is presented in Video S1.

**Figure 5:**
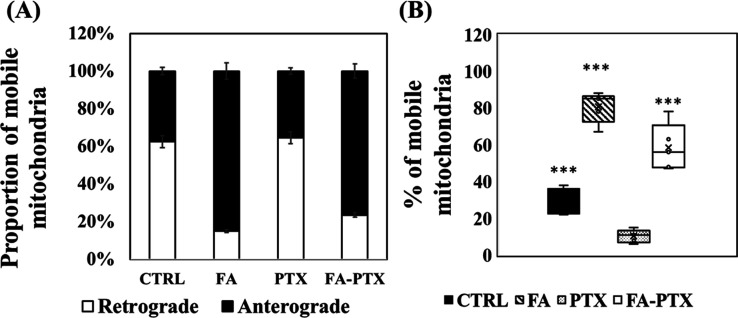
(A) Proportion of anterograde and retrograde mitochondria found in the axonal chamber and (B) percentage of mobile mitochondria to the stationary mitochondria. FA treatment induced a significant increase in mitochondrial mobility compared to the control, while PTX treatment almost completely abrogated mitochondrial mobility. FA-PTX treatment restored mitochondrial mobility. The data represents the respective average values from 20–30 mitochondria; error bars = S.D.; the significant differences in the proportion of motile to stationary mitochondria between PTX or other groups were indicated as ***p < 0.001.

FA treatment alone and in a cotreatment with PTX increased the proportion of anterograde mitochondria (Figure 5A). Similarly, the induction of FA into culture-enhanced motile mitochondria sharply reached up to 82% with respect to stationary mitochondria; this was superior to control levels which had only an average of 28% (Figure 5B). As expected, the PTX exposure alone led the mitochondrial mobility to almost null, but combined treatment with FA resulted in mobile mitochondria which increased again to 45–60% (Figure 5B), clearly indicating that FA facilitated movement from the stationary phases.

## 3 Discussion

It has been well established that chemotherapy is associated with peripheral neurotoxicity.^26,27^ Studies have shown that mitochondrial movement impairment causes the progression of chemotherapeutic-induced neurodegeneration.^25,28^ Previously, our research showed that peripheral axons are vulnerable to PTX therapy, leading to axon degeneration and impairment of outgrowth, while the cell bodies were unharmed during that exposure time,^29^ consistent with the findings of other studies.^30^ PTX treatment for cancer patients results in a decline of the mitochondrial population, health, and interaction of axonal mitochondria to the somal mitochondria.^12,31^ DRG cells of the mice with cisplatin-induced neuropathy had mitochondria with compromised ability for energy production.^32^ However, no established medical treatment that can prevent or cure CIPN/PIPN exists. In anticipation of new drug candidates for the neuroprotection against CIPN, several compounds such as omega-3 fatty acids,^13^ ramipril,^33^ minoxidil (CN016),^9^ and ethoxyquin^34^ have been studied. We have screened 2322 compounds and identified FA as a neuroprotective drug.^16^ FA is a glucocorticoid drug used to treat psoriasis of the scalp and relieve other complications caused by skin conditions.^35^ The FA cotreatment with PTX prevented the suffering of axons of DRG cells from degeneration as assessed by the measurement of axonal length in vitro and neuroprotection by increasing the density of intraepidermal nerve fibers when assessed in in vivo mouse models.^16^ Previously, it was shown that FA cotreatment improved cellular bioenergetics by improving the ATP level significantly in comparison with PTX-treated DRG cells (*P* < 0.01). Glybina and group^17^ have shown that intravitreal infusion of FA is neuroprotective in retinitis pigmentosa, a clinical condition with visual disability due to degeneration of photoreceptor cell death. The recovery of the axon morphology as shown in Figure 1 and increased axon length (Table 1) following FA-PTX cotreatment highlighted the rescuing effect of FA, consistent with the previous report.^16^ Low doses of FA (10 and 20 nM) alone or cotreatments with PTX showed preferably enhanced axon growth and protection compared to FA in the 100–200 nM range. However, FA100 still enhanced axon growth and protection against PTX100. Overall dose–response data suggestion is that low concentration of FA had higher neuroprotection. Herein, achieving the bioavailability of FA within a small window could be a challenge to achieve the best neuroprotection in a clinical application. Results further indicate that FA cotreatment was still effective in neuroprotection during the 5-day test period. Interestingly, the post-FA treatment was also found to be useful in protecting the axonal integrity and still supporting the growth (Figure S2). Glucocorticoids have been known for promoting neuroregeneration by immunosuppression, reducing lipid peroxidation, and increasing myelination.^36,37^ Progesterone, a steroid, exhibits neurotrophic, neuroprotective, antioxidant, and anti-inflammatory effects.^38^ Estradiol, a steroid treatment in rats, is involved in oxidative phosphorylation and increases mitochondrial activity.^39^ However, there is little evidence that other steroids act to enhance biogenetics, and to our knowledge, no study has aimed to study FA-mediated neuroprotection by mitochondrial enhancement.

Improved mitochondrial biogenesis is a well-established model of neurons for high regenerative capacity.^40,41^ Mitochondrial enrichment in axons is significantly altered after chemotherapeutics.^42^ Taken together, herein, we explore neuroprotective mechanisms of FA against PIPN in terms of mitochondrial transport. The effect of PTX/FA/FA-PTX on axonal mitochondria in DRG cells is shown using the proposed two-color staining approach. Creating two chambers connected by straight microchannels ensures a template for typical polarized neuron cell growth, thereby being suitable for the study of neuron-specific subcellular mitochondria. Imaging of the axonal chamber after subcellular staining reveals that there were both axonal mitochondria in red color and somal mitochondria in blue color present, indicating that proximal mitochondria traveled to the distal axons as new recruits. Although the concept of directional mitochondria trafficking and its effect on axonal regeneration has been established in the past,^43,44^ selective identification of the mitochondrial origin and prediction of trafficking of specific mitochondria in a single platform have not been reported so far. It is an improvement compared to contemporary works in which single Mito probes were used to evaluate mitochondrial dynamics. The major advantage of this approach over the conventional approach could be that it allows mitochondria trafficking to be distinguished visually before being analyzed with advanced tools. The possible dye diffusion between the chamber was avoided by maintaining equal medium volume on both sides. We have shown fluidic isolation in compartmentalized chambers in a previous report where there is no diffusion of dyes when allowed for 5 days.^45^ The restriction of dye diffusion can be achieved by physical barriers, such as microgrooves or thin membranes, which separate the compartments while still allowing for the growth of axons through small channels. These barriers prevent the direct contact and diffusion of dyes or drugs between the compartments.^46^

Mitochondrial movement was found to be negligible in a typical PIPN in vitro condition created by exposing DRG cells to PTX. There was evidence that a decrease in mitochondrial dynamics was induced by another tubulin-stabilizing agent, vincristine.^47^ We observed that upon the treatment with FA-PTX, an enhanced mitochondrial population in distal axonal was found (Figure 2B,C) which may be attributed to the beneficial effects of FA on its action of preventing mitochondria dysfunction and preserving the axon quality. Mitochondrial mobility is another important factor in vital neuronal processes.^48^ Mitochondria move bidirectionally along axons and can change mobile and stationary phases in response to changes in metabolic status and growth conditions. Anterograde transport contributes to energy supply and neuronal survival, while retrograde transport helps in the clearance of dysfunctional mitochondria.^44^ Results revealed that PTX-only-treated axons had almost null mitochondrial mobility. The reason could be that PTX induced simultaneous mitochondrial damage and axonal degeneration. Reactive oxygen species formation and hyperstabilization of microtubules in response to PTX are the common causes of mitochondrial impairment.^49^ Interestingly, the cotreatment with FA enhanced the anterograde and retrograde mobility significantly with superior velocity (Figure 5). The outcome predicts that the ability of fast and long anterograde mitochondrial transport is a clear indication of increasing recruitment of mitochondria by FA. Moreover, FA cotreatment with PTX resulted in an increase in mobile mitochondria sharply with respect to stationary mitochondria (Figure 5, *P* < 0.001), suggesting that FA triggers mitochondria for movement. Improving mitochondrial transport in the injured and diseased neurons contributes to neuronal repair.^22,50^ The stronger regenerative capability of axons appears to be positively correlated with greater mitochondrial motility. Xu et al.^51^ showed that the injured axons displayed vigorous regeneration, accompanied by increased mitochondrial motility when treated with dibutyryl cyclic adenosine monophosphate as a mitochondria enhancer. It was found that FA-PTX-treated cells had a higher mitochondrial population in distal axons compared to PTX-treated cells (Figure 2D). This hints that FA could protect the mitochondria. The higher population increment is a logical consequence of increased transport into the distal axons from the soma.

Neurosteroids including progesterone and estradiol are considered effective in having neuroprotective effects in Alzheimer’s disease by increasing ATP production and mitochondrial membrane potentials.^39,52^ Superior ATP production by the FA cotreatment could play the role of neuroprotection against PIPN.^16^ Furthermore, enhanced anterograde mitochondrial trafficking and mobility combinedly might have induced neuroprotection. A previous report revealed that cotreatment of FA-PTX did not affect the tumor cell-killing ability of PTX in in vitro cancer cells.^16^ Microtubule stabilization is the main mechanism of cancer cell killing by PTX.^53^ The differential effects on microtubules induced by PTX and FA are unknown. Therefore, elucidation of the mitochondrial dynamics in cancer cells will be critical for a broad understanding of FA function. Moreover, the role of FA on other chemotherapeutics such as cisplatin, bortezomib, and vincristine-induced peripheral neuropathy in neuroprotection would be a choice for future works. Embryonic rat DRG neuron cells were used in this study, which is a common model system to study the development, function, and disease of adult DRG neurons.^54^ However, CIPN occurs in adult patients treated with various chemotherapeutic agents. Hence, certainly, there are some limitations to using embryonic rat DRG neurons. The embryonic DRG neurons are still developing and have higher plasticity, thereby having different survival and neuron rescuing strategies under cellular stress and injuries from adult neurons.^55^ Adult neurons should be always a choice to increase the generalization of the findings of neuroprotection studies against peripheral neuropathy.

## 4 Materials and Methods

### 4.1 Materials

Polydimethylsiloxane (PDMS) and the SYLGARD 184 Silicon elastomer curing agent were purchased from Dow Chemical Company, USA. Red fluorescent MitoView and blue fluorescent MitoView were obtained from Biotium, USA. Similarly, poly-d-lysine (PDL) and laminin were purchased from Sigma-Aldrich, USA. 100× penicillin/streptomycin (P/S), 100× glutamate, B27 supplement, 20 ng/mL glial-derived nerve growth factor (GDNF), and 5-fluoro-2′-deoxyuridine thymidylate synthase inhibitor (FUDR) were received from Sigma-Aldrich, USA. PTX and FA were obtained from Alfa Aesar, USA. All other chemicals were of analytical grade and used as received.

### 4.2 Preparation of the Compartmentalized Microfluidic Culture System

The compartmentalized chamber system developed is a roughly measured PDMS rectangle with two chambers connected with 10 μm wide and 500 μm long microchannels each spaced 35 μm apart. The device was prepared via a high-resolution photolithographic process as detailed in the previous report.^29^ In brief, PDMS and the curing agent were mixed thoroughly in a 10:1 weight ratio, followed by the removal of bubbles by a desiccator (SP Scienceware, USA). Then, the mixture was poured into a silicon mold and placed in a hot oven at 85 °C for 2 h to cure. The selective segment of channels was created by using a biopsy puncher (Huot Instruments, Michigan) 6 mm in length and 4 mm in width with shape modification. The as-prepared microfluidic device was bonded to glass slides (0.15 mm thickness) by plasma treatment (Cute-MP, Femto Science, South Korea). The microchannel bonded devices were sterilized by autoclaving before the cell culture work started. Later, the chambers were coated with 100 μg/mL PDL and 5 μg/mL laminin overnight at 4 °C followed by washing prior to cell seeding.

### 4.3 DRG Cell Extraction and Culture

All experiments related to animals were conducted in accordance with protocols approved by the Institutional Animal Care and Use Committee (IACUC) of the National University of Singapore. The DRG cells were collected from the embryos of an E15 pregnant Sprague Dawley rat after they were euthanized under an isoflurane flow of 7.5 L/min according to our recent works.^56^ To collect the DRG cells, the incision site was sterilized with 70% ethanol on the dorsal region of the abdomen and an incision was made to expose the abdominal cavity. The uterus was then removed and detached from the cervix and ovaries and placed in L-15 media with 1% P/S. The embryo was dissected under a dissection microscope (Nikon SMZ745T, Japan) with a high-density illuminator (Fiber-Lite, M1-150, Dolan-Jenner Industries, USA). The DRG cells were collected by dissecting the spinal column and placing them into another dish containing fresh 1 mL of L-15 media with 1% P/S. Later, the cell suspension was prepared with 0.25% trypsin for 5 min at 37 °C, quenched with the fresh medium, and centrifuged to collect the DRGs. The cells were then resuspended in a DRG cell culture medium at a density of 50,000 cells/mL and seeded on each soma well with a density of 5000 cells in 100 μL of the medium unless otherwise stated and allowed for 30 min at 37 °C to attach. The neurobasal medium supplemented with 1% 100× P/S, 100× glutamate, B27 supplement, and glial-derived 20 ng/mL nerve growth factor (GDNF) was used for the DRG cell culture. 13 μg/mL FUDR was also added until 48 h of plating for the elimination of non-neuronal glial cell contamination.^57^ The cells were maintained by replenishing the half-well media every 48 h.

### 4.4 Effect of FA-PTX on Axon Lengths

The neuroprotective role of FA in PTX-treated DRG cells was studied in terms of axon length changes in response to FA, PTX, or their combination. The E15 DRG cells with 1000 cells were cultured on PDL/laminin-coated 96-well plates for 24 h. 2.5 mM PTX was prepared as a stock by dissolving PTX in a Kolliphor EL/ethanol (50/50) solvent by weight and stored at −20 °C. FA was then dissolved in the Kolliphor EL/ethanol 50/50 solvent by weight, and a stock concentration of 3 mM was made and maintained at −20 °C. FA (10, 20, and 100 nM), PTX (10, 50, 100, and 200 nM), and a combination of FA/PTX were added onto 24 h cultured cells, and plates were further incubated for another 24 h. The cells were stained with a 2 μM calcein-AM (Corning, USA) dye-containing medium followed by live-cell imaging by a fluorescence microscope (Leica DMI8, Germany). Image J Fiji [ImageJ 1.53t, Java 1.8.0-345 (64-bit)] software was used to estimate the length of the axons. At least 150 cells were used for calculating the average axon length for each group. Triplicate experimental samples were used in each group.

### 4.5 Evaluation of the Effect of FA-PTX on Mitochondria of DRG Cell Cultures

DRG cells were cultured in the soma of the compartmentalized system as mentioned above. It was found that axons traversed the microchannels at 5 days of culturing, a consistent observation as reported earlier.^58^ DRG cell culture (both soma and axons) was treated with 100 nM PTX with/without FA in neurobasal media along with B27 supplement with antioxidants and 1× GlutaMAX. The drug-treated culture was incubated for 24 h before adding MitoTracker in somal and axonal chambers. The axonal chamber was studied microscopically. Different concentrations, 200 nM/200 nM, 200 nM/300 nM, and 200 nM/400 nM of red/blue MitoTrackers, were used for the optimization study. The concentration of dyes was chosen based on the suggested range by the manufacturer’s instructions. A dye optimization study revealed red and blue mitochondria observed in the axonal chamber clearly (Figure S4). The mitochondria signals are independent of the concentrations of the MitoTracker. Microscopy configurations were set as follows, using a Nikon 63× Fluor-view oil-immersion lens at an imaging speed of 9 with a 512 × 512 pixel resolution with the laser intensity set at 0.2% for a 5 mW red laser with a 650 V master gain and a 49 μm aperture size with 8 averaged frames giving a 5.03 s temporal resolution per captured frame for dual photon imaging. The imaging provides the following information, as shown in Table 2, after subsequent data processing.

**Table 2:** Mitochondrial Trafficking

S.N	test output
1	number of proximal mitochondria (PM) and distal mitochondria (DM)
2	velocities of both PM and DM
3	percentages of stationary and mobile mitochondria

### 4.6 Quantification of Somal vs Axonal Mitochondria

For the counting of the somal and axonal mitochondria, both chambers should be separated a little to avoid cell migration between the chambers at the initial stage of seeding. The somal mitochondria were dyed with MitoTracker blue, while the axonal compartment was dyed with MitoTracker red. Later, the axonal chamber was studied microscopically to determine the engagement of the somal mitochondria to the axonal side. This enabled the site-specific tracking of the mitochondrial origin either from the somal or axonal sides. The total number of mitochondria labeled with MitoTracker red/blue was determined automatically via open-source software Fiji’s “Find Maxima” function with a noise tolerance of 25. After denoising the video via the “Despeckle” function, the number from one dye condition from each image was compared against the total number of mitochondria.

To quantify the mobile mitochondria, the time-lapse video was then converted to the default 8-bit binary mode. The number of mitochondrial tracks was obtained with the aid of a “Difference Tracker” plugin in Fiji which has a comparatively accurate number of mitochondrial tracks with respect to other automated tracking methods.^59^ The “Difference Filter” function was used to obtain the reference differential video to track any moving mitochondria with the minimum difference filter = 12 and the difference frame offset = 2, while the “Mass Particle Tracker” function was used to count the number of mobile tracks with the following parameters: minimum tracked intensity = 20, minimum feature size = 2, initial flexibility = 25, subsequent flexibility = 40, and minimum track length = 4. The output was taken from the total track counts as the total number of moving mitochondria. Alternatively, mitochondria numbers were crosschecked by manual counting.

### 4.7 Quantification of Mitochondrial Motility in DRG Cells

The mitochondrial motility was measured via determination of mitochondrial velocity across different frames via a fixed temporal resolution of 5.03 s between frames and 0.195 μm/pixel for a 512 × 512 pixel resolution. To quantify the mitochondrial velocity, live images were analyzed with the aid of the ImageJ/Fiji^60^ and the movies obtained were aligned using the “StackReg” and “TurboReg” algorithms which accommodated for any stage drift first and denoised with the despeckle function in Fiji. The actual velocity was obtained with the aid of the “MTrackJ” plugin for manual tracking of individual mitochondria across the frames until the mitochondria disappear, stop moving, or move out of frame. The following parameters were obtained from the analysis from MTrackJ, orientation and average velocity of moving mitochondria. Two anomalous situations which hindered tracking of motile mitochondria were “pauses” and “set-backs” being defined as intervals where the mitochondria moved less than 2 pixels (which arise due to “jittery” conditions from manual tracking, wherein the ends of the mitochondria dimmed or resumed motion at the same position) and phases where mitochondria briefly appeared, disappeared, and reappeared further along the axon in the same trajectory, respectively.

Quantification of direction-specific mitochondrial migration was completed with the aid of a comparison of progressive *y*-axis coordinates between tracking points as the videos were always orientated in a top-down anterograde direction. Anterograde trafficking is represented in the upper-left to the lower-right directions, while retrograde trafficking is represented in the opposite direction. Stationary mitochondria are represented as columns with respective colors.

### 4.8 Statistical Analysis

All data sets were presented as a mean ± standard deviation except where noted. Each group was made in triplicate. The probability value (*P*-value) between the groups was analyzed by the two-tailed *t*-test provided in Microsoft Office Excel 2022 unless otherwise stated. A *P*-value of less than 0.05 is considered statistically significant.

## 5 Conclusions

In the present study, FA has shown its capability of axonal protection following FA-PTX cotreatment and post-FA treatment. Furthermore, FA treatment also attenuated the mitochondrial bioenergetics and mitochondrial transport in the axons of DRG cells. Treatment with FA enhanced the anterograde mitochondrial trafficking and velocity remarkably compared to PTX-treated conditions. The two-color mitochondrial staining approach was found applicable to test the mitochondrial transport mechanism in DRG cells under different drug responses. Taken together, this study provides a novel insight into the mechanisms underlying FA-induced neuroprotection against PIPN and insists that FA could be a therapeutic drug having the potential to prevent peripheral neuropathies. This study also addresses the concerns of having difficulties in tracking axonal transport by putting forth a strategy of a two-color staining approach in a compartmentalized chamber.

## Supporting Information Available

Effect of drugs on DRG cells with 5-day treatment; effect of post-FA treatment on axon lengths; mitochondrial images under FA-PTX cotreated conditions at 48 h; and MitoTracker concentration optimization study (PDF)
Mitochondrial trafficking under FA-PTX treatment (AVI)

## Supplementary Material

cn3c00218_si_001.pdf

cn3c00218_si_002.avi
