# Supplementary material for: Fluocinolone Acetonide Enhances Anterograde Mitochondria Trafficking and Promotes Neuroprotection against Paclitaxel-Induced Peripheral Neuropathy
Source: ACS Chem Neurosci. 2023 May 11;14(11):2208–16. doi: 10.1021/acschemneuro.3c00218 (PMC10251481; doi:10.1021/acschemneuro.3c00218)
Supplement: Supplementary file 1 — cn3c00218_si_001.pdf [file cn3c00218_si_001.pdf]

Supporting information for

Fluocinolone Acetonide Enhances Anterograde Mitochondria Trafficking and Promotes Neuroprotection Against the Paclitaxel Induced Peripheral Neuropathy

Arjun Prasad Tiwari[1,] Lee Ji Chao Tristan[2,3], Bayne Albin[1], In Hong Yang[1]*

[1]Department of Mechanical Engineering and Engineering Science, Center for Biomedical Engineering and Science, University of North Carolina at Charlotte, Charlotte, North Carolina 28223, United States

[2]Department of Biomedical Engineering, National University of Singapore, Singapore

[3]School of Medicine, University of Western Australia, Perth, Western Australia 6009, Australia

* Corresponding authors: In Hong Yang; E-mail: iyang3@uncc.edu

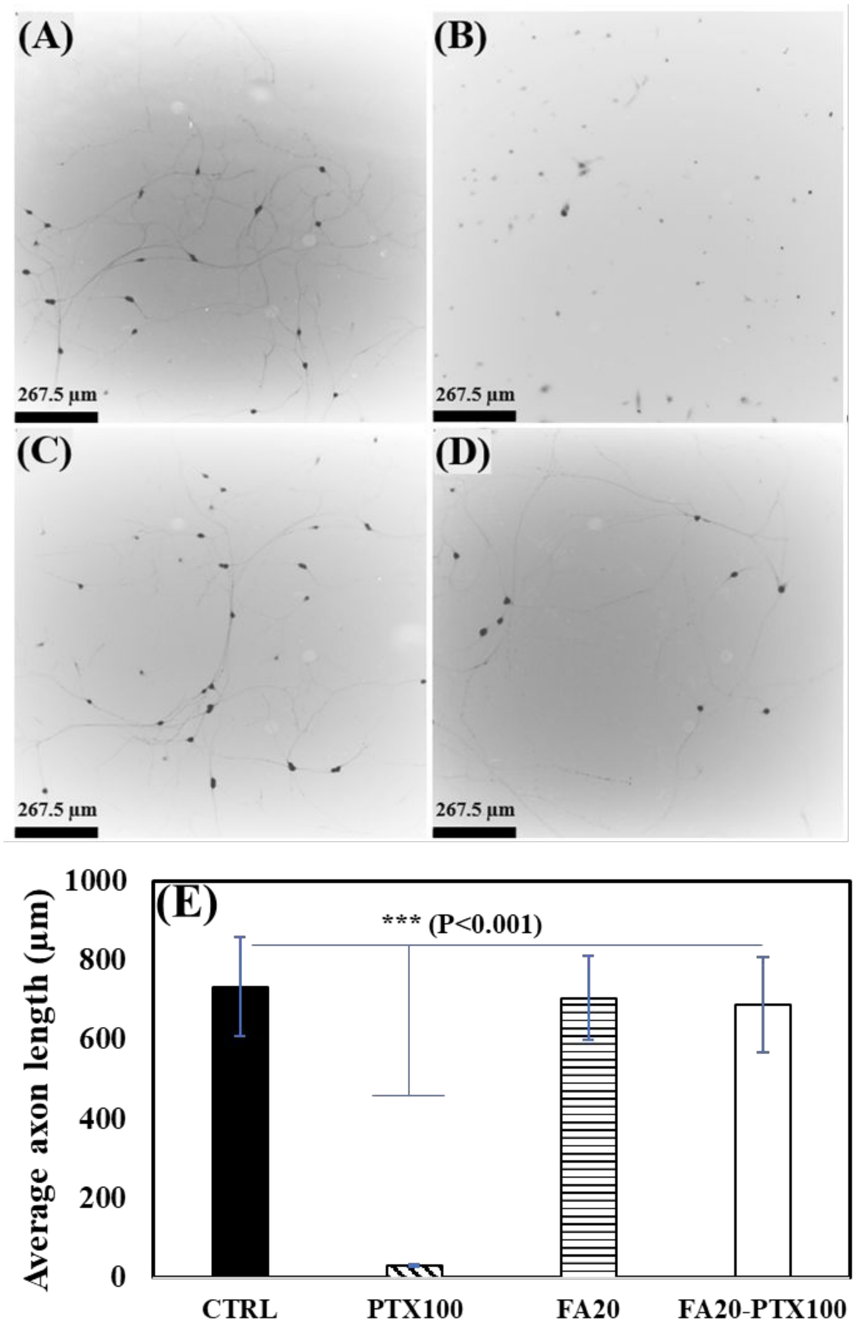

Figure S1. *The effect of drugs on DRGs with 5-days treatment. Representative calcein stained florescence images of the 5-days drugs treated DRGs; Control (A), B(PTX), C (FA) and FA-PTX, and (E) corresponding axon length data. The 500 cells were seeded in each 96-well followed by treatment at 24 h point and allowed another 5-days before imaging. Media containing respective drugs was replenished 50% by volume at 48 h interval. *** represents the significance between the PTX100 and other samples (P<001).*

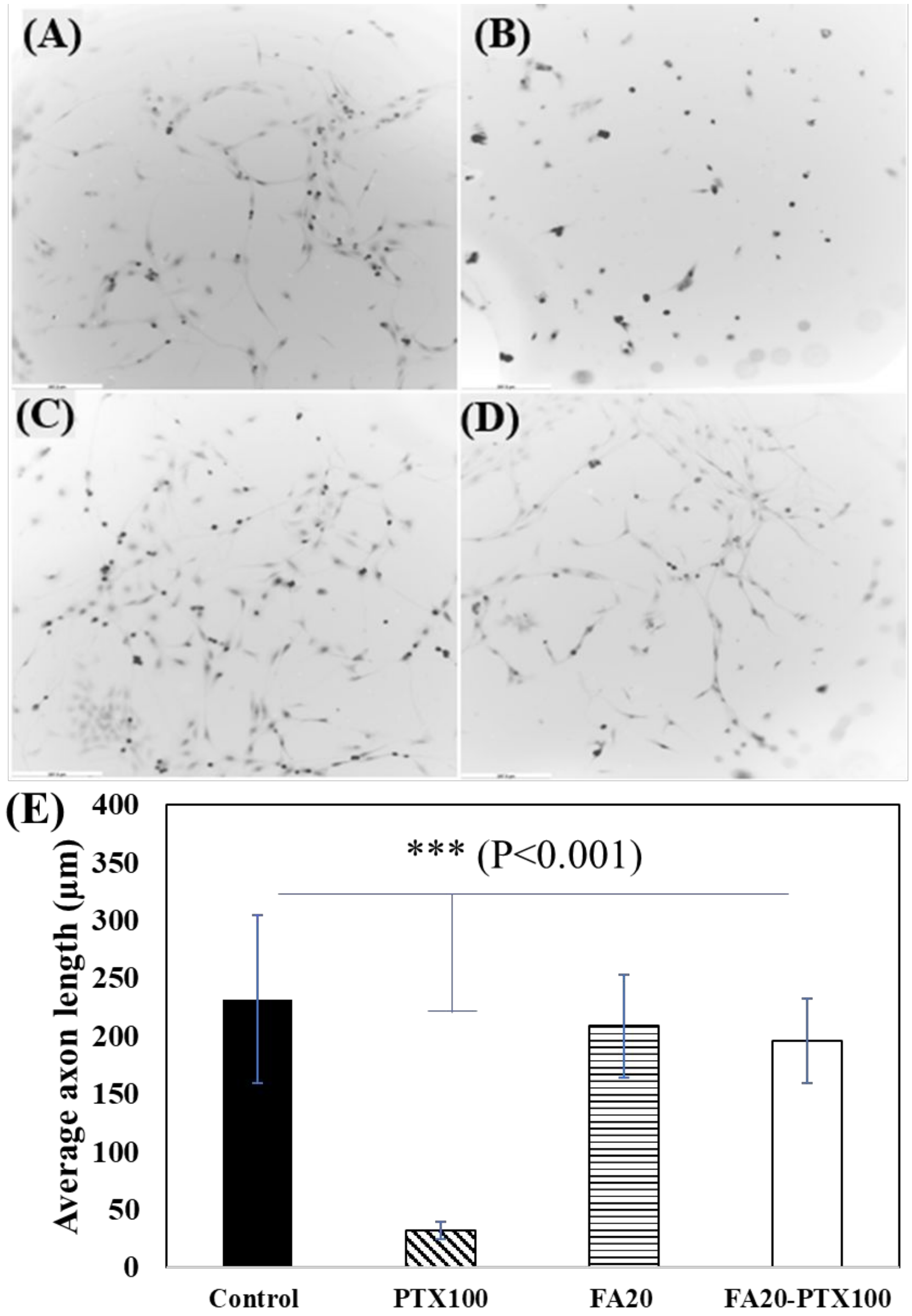

Figure S2. *The effect of post FA treatment on axon lengths, A(control), B (PTX100), C (FA20-PTX100) and D (FA20) and (E) corresponding axon length data. *** represents the significance between the PTX100 and other samples (P<001).*

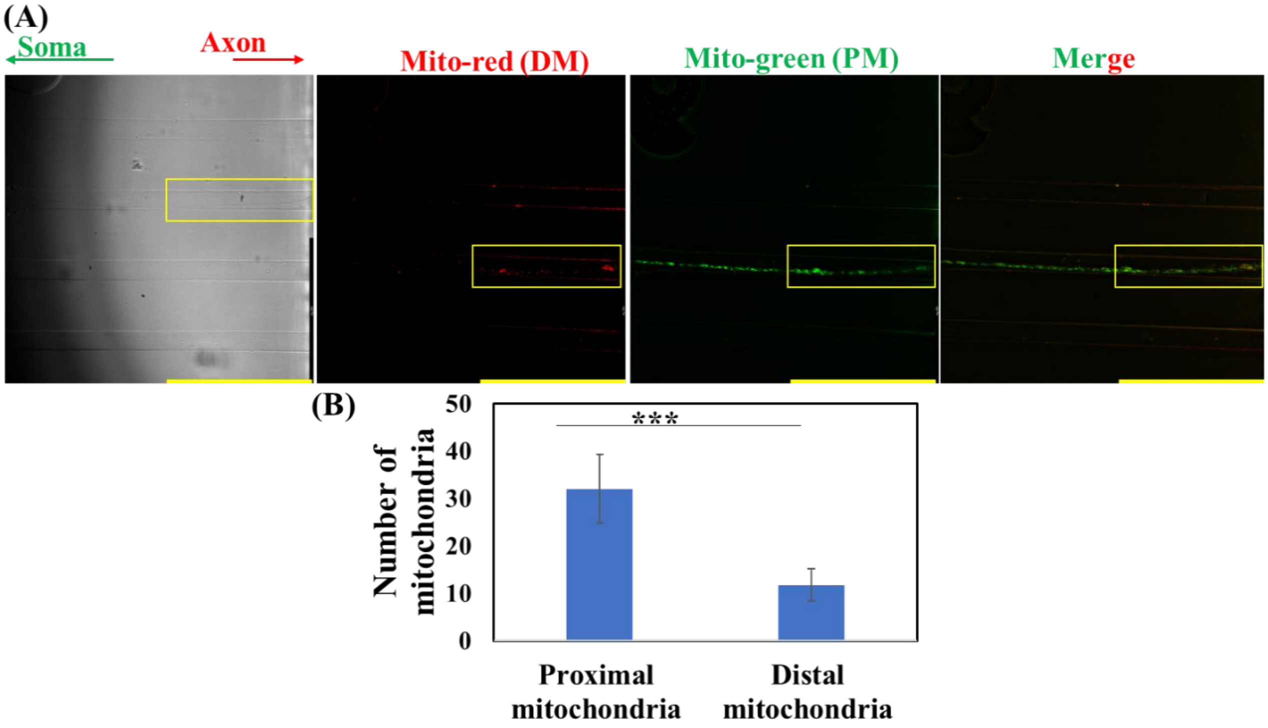

Figure S3. *(A) Mitochondrial images under FA-PTX cotreated condition at 48 h. Mito-tracker green was used to stain the soma mitochondria while Mito-tracker red was used to stain the axonal mitochondria. The imaging was taken close to the axonal chamber. Scale bar is 100µm. (B) a graph showing the average number of mitochondria corresponding to Figure A. *** represents the significance between the proximal and distal mitochondria (P<001).*

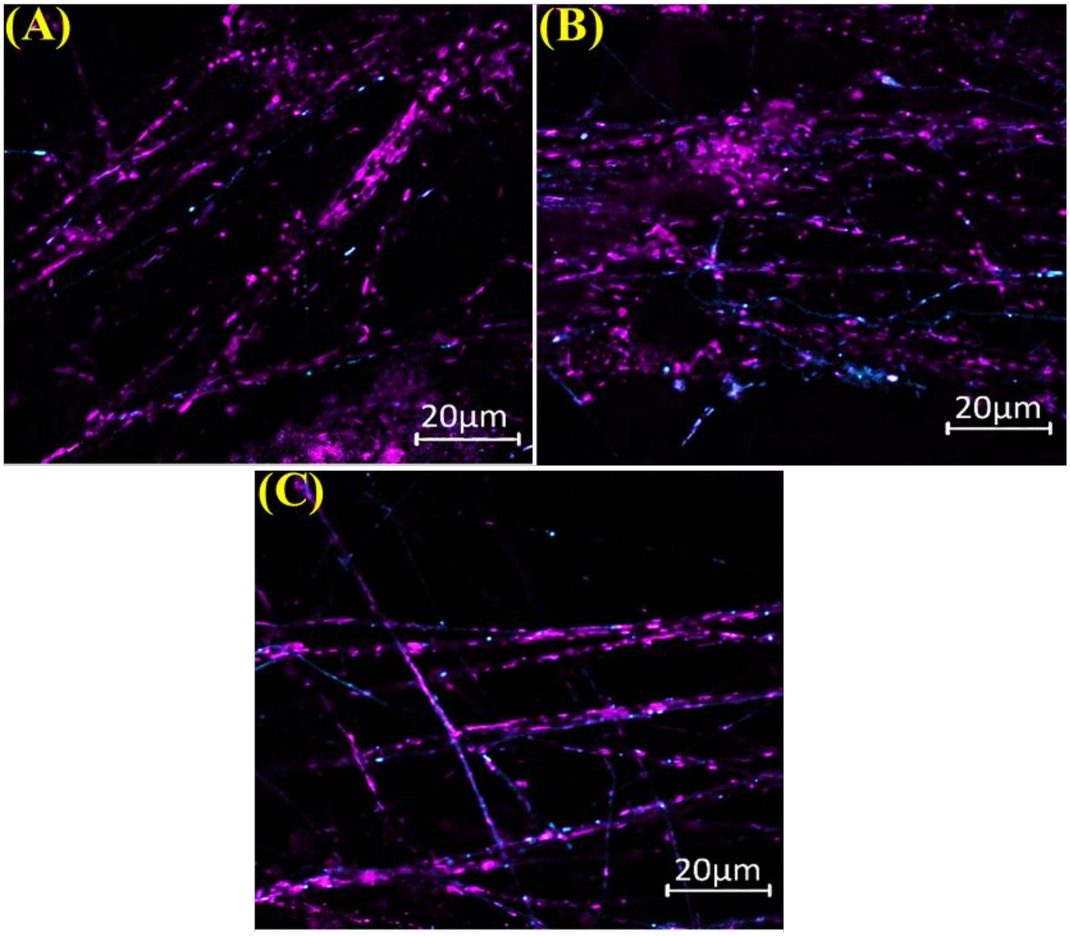

Figure S4. *MitoTracker concentration optimization study; DM were labeled with MitoTracker Red keeping constant at 200 nM while PM were stained with varying Mitotracker blue concentrations; 200 nM (A), 300nM (B), and 400 nM (C).*

S-Video1- *Mitochondria trafficking under FA-PTX treatment. Imaging was taken at axonal chamber.*
